# Diffusion-Weighted Magnetic Resonance Imaging in Hepatocellular Carcinoma as a Predictor of a Response to Cisplatin-Based Hepatic Arterial Infusion Chemotherapy

**DOI:** 10.3389/fonc.2020.600233

**Published:** 2020-11-19

**Authors:** Pil Soo Sung, Moon Hyung Choi, Hyun Yang, Soon Kyu Lee, Ho Jong Chun, Jeong Won Jang, Jong Young Choi, Seung Kew Yoon, Joon-Il Choi, Young Joon Lee, Si Hyun Bae

**Affiliations:** ^1^The Catholic University Liver Research Center, College of Medicine, The Catholic University of Korea, Seoul, South Korea; ^2^Division of Gastroenterology and Hepatology, Department of Internal Medicine, College of Medicine, Seoul St. Mary’s Hospital, The Catholic University of Korea, Seoul, South Korea; ^3^Department of Radiology, College of Medicine, Eunpyeong St. Mary’s Hospital, The Catholic University of Korea, Seoul, South Korea; ^4^Division of Gastroenterology and Hepatology, Department of Internal Medicine, College of Medicine, Eunpyeong St. Mary’s Hospital, The Catholic University of Korea, Seoul, South Korea; ^5^Department of Radiology, College of Medicine, Seoul St. Mary’s Hospital, The Catholic University of Korea, Seoul, South Korea

**Keywords:** hepatocellular carcinoma, hepatic arterial infusion chemotherapy, magnetic resonance, diffusion restriction, objective response

## Abstract

This study aimed to identify the utility of diffusion-weighted magnetic resonance (MR) imaging with an apparent diffusion coefficient (ADC) map as a predictor of the response of hepatocellular carcinoma (HCC) to cisplatin-based hepatic arterial infusion chemotherapy (HAIC). We retrospectively evaluated 113 consecutive patients with Barcelona Clinical Liver Cancer (BCLC) stage B or C HCC, who underwent gadoxetic acid-enhanced and diffusion-weighted MR imaging. The appropriate cutoff for the pretreatment tumor-to-liver ADC ratio was determined to be 0.741. Of the 113 patients, 50 (44%) presented with a pretreatment tumor-to-liver ADC ratio < 0.741 (low group). Evaluation of the treatment response after 2-3 cycles of HAIC in these 50 patients revealed that 21 patients (42%) experienced an objective response to HAIC. On the other hand, only 11 of the 63 patients (17%) with a pretreatment tumor-to-liver ADC ratio ≥ 0.741 (high group) showed an objective response. Thus, the objective response rate was significantly higher in the low group than in the high group (*P* = 0.006). Multivariate logistic regression analysis using parameters including perfusion alteration, percentage of non-enhancing portions, and pretreatment tumor-to-liver ADC ratio revealed that a pretreatment tumor-to-liver ADC ratio < 0.741 (odds ratio 3.217; *P* = 0.014) was the sole predictor of an objective response to HAIC. Overall survival rates were significantly higher in patients with objective responses to HAIC than in those without objective responses (*P* = 0.001 by log-rank test). In conclusion, patients with BCLC stage C or C HCC with a pretreatment tumor-to-liver ADC ratio < 0.741 showed a favorable intrahepatic response to cisplatin-based HAIC. Therefore, diffusion-weighted MR imaging can play a critical role as a predictor of response to cisplatin-based HAIC in unresectable HCC.

## Introduction

Hepatocellular carcinoma (HCC) is the fourth most common cause of malignancy-related death worldwide (1). A considerable number of patients with advanced HCC receive only palliative treatments in East Asian countries, where hepatitis B virus (HBV) infection is prevalent and accounts for more than 70% of the patients (2). To enhance survival outcomes, sorafenib and lenvatinib are usually administered in cases of advanced HCC with portal vein tumor thrombus (PVTT) or extrahepatic metastasis. However, these drugs only have modest treatment responses and may even have notable side effects (3, 4). Furthermore, the latest immune checkpoint inhibitor monotherapy did not demonstrate increased survival compared with sorafenib in patients with unresectable HCC (5).

Barcelona Clinical Liver Cancer (BCLC) stage B or C HCC cases with high intrahepatic tumor burden can alternatively be treated through hepatic arterial infusion chemotherapy (HAIC), whereby the drug is administered directly through a port inserted into the liver. HAIC enables higher drug concentration in intrahepatic tumors with minimal systemic adverse effects (2). There is research evidence that, compared to sorafenib, both the objective response and survival outcomes are improved when advanced HCC is treated through HAIC (2, 6, 7). Moreover, recent studies demonstrated that significant reduction of the intrahepatic tumor by HAIC in HCC with PVTT and/or extrahepatic metastases led to better survival outcomes than no reduction of the intrahepatic tumor burden (2, 8). Therefore, in advanced HCC, it is crucial to identify patients who will potentially benefit from HAIC before start the treatment.

A non-invasive diagnosis of HCC is established by a characteristic radiological findings of arterial phase hyperenhancement (APHE) and portal venous or delayed phase “washout” on contrast-enhanced computed tomography (CT) or magnetic resonance imaging (MRI) (9). Recently, a hepatocyte-specific contrast agent, gadolinium-ethoxybenzyl-diethylenetriamine penta-acetic acid (Gd-EOB-DTPA, gadoxetic acid), has been recognized as the critical tool for the detection of early HCCs. Moreover, diffusion-weighted imaging (DWI) obtained during MR examination was reported to estimate the biological behavior of HCC (10, 11). In general, DWI depends on the information of the diffusivity of water molecules, reflecting the cellularity of the tumors (12). DWI has a potential for use in various liver diseases (13). It can be used as a biomarker for liver fibrosis, HCC detection, and predicting responses to anticancer therapies (14). DWI is an attractive technique in liver diseases because it may add qualitative and quantitative information to conventional MRI sequences and it can be easily performed (13).

Evaluation of HCC treatment responses based on the detection of features in DWI MR has been undertaken by several studies. Previous reports showed that pretreatment apparent diffusion coefficient (ADC) of HCC can be predictive of response to transarterial chemoembolization (TACE) (15, 16). Moreover, ADC change relative to baseline (ADC ratio) 1 month after TACE was an independent predictor of progression-free survival in HCC (17). However, there are no reports that describe the association between various MR parameters and responses to HAIC in HCC. In this study, we aimed to identify the utility of various MRI findings and DWI with an ADC map as a predictor of the intrahepatic response of HCC to cisplatin-based HAIC.

## Methods

### Study Design and Population

**Figure 1:**
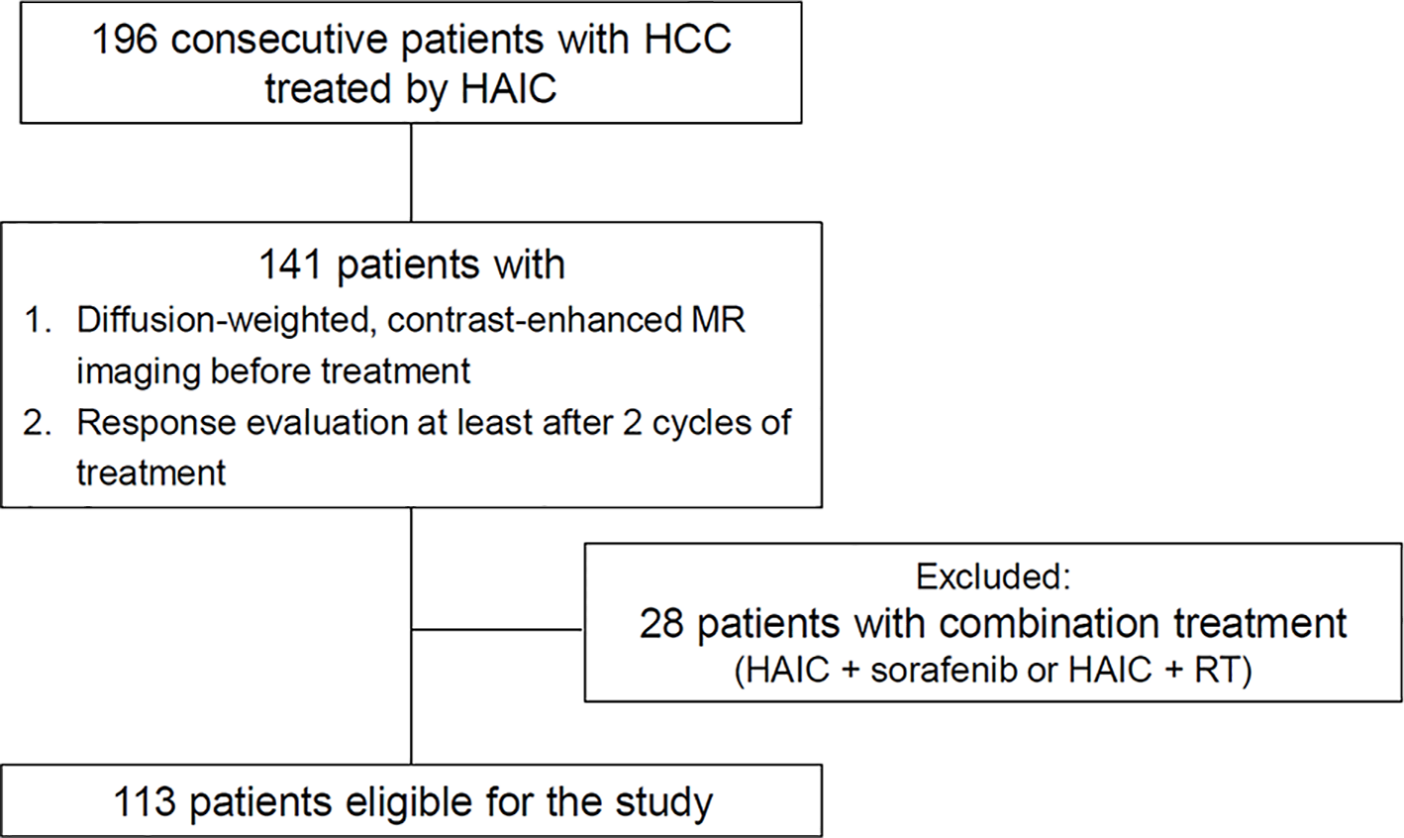
Study population.

Ethical approval was obtained from the Institutional Review Board of Seoul St. Mary’s Hospital (KC19RESI0912). A diagnosis of HCC was confirmed in every enrolled patient by the updated international guidelines (9, 18, 19). HCC cases with PVTT or infiltrative tumors are occasionally treated with HAIC rather than sorafenib or lenvatinib in the researcher’s institution. The medical records of all cases that received an HCC diagnosis between January 2010 and December 2017 were reviewed by experienced hepatologists. The survival data of the patients continued to be followed up until December 2019. The inclusion criteria of this study were as follows: unresectable HCC cases with Barcelona Clinical Liver Cancer (BCLC) stage B or C undergoing HAIC monotherapy, age range of 20–80 years, Child-Pugh class A or B, Eastern Cooperative Oncology Group (ECOG) performance status of less than 2, lack of indication of bone marrow inhibition (white blood cell ≥ 3000/µL, hemoglobin ≥ 8 g/dL, and platelet count ≥ 7.5×10^4^/µL), normal renal function with levels of serum creatinine < 2.0 mg/dL, diffusion-weighted, contrast-enhanced MR imaging before treatment, and response evaluation at least after 2 cycles of treatment. The study did not include cases in which HAIC was undertaken after sorafenib administration. Patients without response evaluation or with combination treatments (HAIC + sorafenib or HAIC + radiation therapy) were excluded from the analyses. Finally, 113 patients were enrolled in this study (**Figure 1**).

### Diagnosis of HCC

Multiphasic CT, MRI, biochemical analysis of alpha-fetoprotein (AFP), and additional biomarkers provided diagnostic information for HCC. The modified RECIST criteria were used to assess therapeutic response (3). Tumors without arterial enhancement of the target lesions were determined to be in complete response (CR). Partial response (PR) was defined as a 30% reduction in the sum of viable target lesion diameters. Progressive disease (PD) was identified if augmentation of at least 20% was noted in the total viable target lesion diameters. If the findings were outside these definitions, the tumor response was considered a stable disease (SD). RECIST rather than mRECIST was used in cases of infiltrative HCCs because this type is classified by mRECIST as a non-target lesion. Likewise, during the assessment of tumor response, PVTT was not included because it was classified by mRECIST as a non-target lesion.

The Vp stages were employed to categorize the PVTT. Tumor invasion distal to the second portal vein branch was categorized as Vp1, tumor invasion of the second portal vein branch was categorized as Vp2, tumor invasion of the first portal vein branch was categorized as Vp3, and tumor thrombus occurrence in the main portal vein trunk or a branch of the portal vein contralateral to the main affected lobe was categorized as Vp4 (2).

### HAIC Protocol

The specific protocol of cisplatin-based HAIC has been described previously (2). Every HAIC process was conducted by two or more interventional radiologists with more than five years of experience. Two chemotherapeutic drugs were infused through the chemoport inserted into the femoral artery, 5-fluorouracil (5-FU) (500 mg/m^2^) for three days and cisplatin (60 mg/m^2^) on the second day. In cases where the disease did not progress or the therapy did not have severe complications, HAIC was repeated at the interval of 4–6 weeks. The Child-Pugh categorization was applied at every cycle to assess hepatic function, while follow-up multiphasic CT or MRI was applied after 2–3 therapy cycles to assess the response to therapy.

### Qualitative and Quantitative Analyses of MR Imaging

A 3-T MR system (Verio, Siemens Healthcare, Erlangen, Germany) alongside a phased array coil with 8 channels serving as the receiver coil was employed for MR imaging. Breath-hold half Fourier Acquisition single-shot turbo spin echo, respiratory-triggered fast spin echo T2-weighted image with fat suppression and 3D T1-weighted in- and opposed-phase gradient echo with two-point Dixon reconstruction were obtained as previously described (16). Meanwhile, contrast-enhanced study was performed using fat-suppressed 3D spoiled gradient-echo volume interpolated breath-hold examinations. After acquisition of unenhanced images, Gd-EOB-DTPA was injected with a dosage of 0.1mL/kg body weight and a flow rate of 1 mL/s through the antecubital vein followed by a 20-mL saline flush. Arterial phase (30- to 35-second delay), portal venous phase (65- to 85-second delay), transitional phase (3-minute delay), and hepatobiliary phase (HBP) (20-minute delay) were acquired as previously described (20). DWI with echo planar imaging using b values of 0, 50, 500, and 800 s/mm^2^ were obtained and ADC maps were automatically generated using DWI with b values of 0 and 800 s/mm^2^ (20). MR imaging sequences and parameters are presented in **Table 1**.

**Table 1:** MR imaging sequences and parameters.

Parameters	Sequence				
	HASTE T2WI	Fast Spin Echo T2WI	T1-weighted in/opposed phase	T1-weighted 3D GRE	DWI
TR (ms)	600–1000	2000–6000	170–220	2.8–3.5	3500–4200
TE (ms)	80–140	100–140	2.6/1.3	1–1.2	40–50
Flip angle (°)	138	150–160	50–70	11	90/180
Slice thickness (mm)	6	6	6	2	8
Reconstruction interval (mm)	6	6	6	2	8
Acquisition matrix	320–400 × 150–180	380–450 × 180–220	250–300 × 120–170	256 x 156	140–160 × 90–120
Signal averages	1	1	1	1	5
b-values (s/mm^2^)	N/A	N/A	N/A	NA	0, 50, 500

TR, repetition time; TE, echo time; HASTE, half-Fourier acquisition single-shot turbo spin-echo; T2WI, T2-weighted image; 3D, three-dimensional; GRE, gradient echo; DWI, diffusion weighted image; N/A, not assessed.

In this study, several radiologists discussed the methods to quantify the ADC values of tumors, background livers, and spleen. They made agreements how to quantitate ADC values. The data from the patients, which were used in the analyses, were obtained from one experienced radiologists (more than 10 years of experience in abdominal radiology) among the involved radiologists. She recorded the number of tumors (single or multiple), the largest diameter on axial and coronal images, presence of portal vein tumor thrombus, proportion of non-enhancing portion (< 50% or ≥ 50%), perfusion alteration, targetoid enhancement, blood product in tumor, fatty change in tumor, diffusion restriction, tumor signal intensity on arterial phase, and homogeneous enhancement on arterial phase. Definition of the most imaging findings were based on the Liver Imaging - Reporting and Data System (LI-RADS). Perfusion alteration is change from the usual blood supply in the liver parenchyma and we evaluated the presence of regional perfusion alteration near the tumor. Targetoid enhancement is target-like imaging morphology with concentric arrangement of internal components. Blood product is seen as high signal intensity on T1 weighted images of MRI. Intra-lesion fat is increased fat within the tumor and we evaluated it on in and opposed phases of MRI.

APHE is enhancement in arterial phase and enhancing part must be higher than liver parenchyma. So, signal intensity or density of tumor on arterial phase was compared to the liver parenchyma and divided into three categories (higher, similar and lower). Arterial phase enhancement that is most pronounced in periphery (rim APHE) is atypical feature of HCC, homogeneity of arterial enhancement was evaluated whether the arterial enhancement in the tumor was prominent in periphery or not.

Quantitative measurement was undertaken regarding ADC values in lesions and circumscribing healthy parenchyma. Delineation of a region of interest was done on ADC maps for both healthy liver parenchyma and HCCs, steering clear of necrotic and cystic zones, artifacts, and blood vessels within the liver as far as manageable. The regions of interest were drawn with similar size (3 cm^2^) in both of tumors and healthy parenchyma and peripheral portion of the tumor was not included because of frequent partial volume artifact. The region of interest was drawn in the largest tumor in the patients with multiple tumor (20). Ratio of pretreatment tumor-to-liver ADC (tumor ADC/liver ADC) was calculated in each patient.

### Statistical Analysis

Statistical analysis was performed using SPSS version 26 software (IBM Corp., Armonk, NY, USA). The chi-square test was used to evaluate discrete variables from the two cohorts. An independent t-test was employed to compare continuous variables between the two groups. To determine the optimal cut-off value in discriminating objective responses by mRECIST, receiver operating characteristic (ROC) curves were generated for pretreatment tumor-to-liver ADC ratio (**Supplementary Figure 1**). Sensitivity, and specificity were calculated by the optimal cut-off value. Statistical significance was defined as *P* < 0.05. The Kaplan-Meier technique was adopted to estimate the overall survival (OS), and the log-rank test was applied for OS comparison. Determinants of objective responses were identified by conducting multivariate analysis alongside a logistic regression model.

## Results

### Baseline Characteristics

The baseline clinical characteristics of the enrolled patients are listed in **Table 2**. We divided all included patients into two groups according to the pretreatment tumor-to-liver ADC ratio. The cutoff value of the pretreatment tumor-to-liver ADC ratio (0.741) was determined by the receiver operating characteristic (ROC) curve. The patients were divided into two groups: patients with a pretreatment tumor-to-liver ADC ratio < 0.741 (n = 50, low group) and a pretreatment tumor-to-liver ADC ratio ≥ 0.741 (n = 63, high group). There were no differences in sex and etiology of HCC between the two groups, although patients in the low group tended to be younger (**Table 2**). There were no statistical differences in the maximal diameter, tumor number, and the presence of PVTT between the two groups. There were no differences in BCLC stages between the two groups. Most of the patients in both groups were classified as BCLC-C stages. Liver function measured by Child-Pugh class were not significantly different between two groups. A considerable number of patients in both groups received other modalities of treatment (TACE, liver resection, RFA, or sorafenib) before HAIC. Regarding tumor markers, there was no statistical difference in serum AFP levels between the two groups. Before HAIC, the mean tumor ADC (unit, 1.10 ± 0.29 × 10^-3^ mm^2^/s) of the high group was not significantly different from that of the low group (unit, 1.10 ± 0.31 × 10^-3^ mm^2^/s), although the mean pretreatment tumor-to-liver ADC ratio was significantly lower in the low group (*P* < 0.001) (**Table 2**). Moreover, the mean pretreatment tumor-to-spleen ADC ratio was also significantly lower in patients in the low group (*P* < 0.001), although spleen ADC values were not significantly different between two groups. For the other parameters detected in MR imaging (the amount of the non-enhancing portion, the presence of perfusion alteration, the presence of targetoid enhancement, the presence of blood product in the tumor, the presence of fatty change in the tumor, and the tumor signal on arterial phase), there were no statistical differences in these parameters between the low and high groups.

**Table 2:** Clinical parameters of study patients.

Variables	ADC ratio < 0.741		ADC ratio ≥ 0.741		*P*
	(n = 50)		(n = 63)		
	No.	%	No.	%	
Age					0.02
<60	36	72	31	49	
≥60	14	28	32	51	
Sex					0.074
Male	44	88	47	75	
Female	6	12	16	25	
Etiology					0.096
HBV	46	92	46	73	
HCV	1	2	7	11	
Alcohol	1	2	6	10	
HBV + HCV	0	0	1	2	
Others	2	4	3	5	
BCLC stage					0.533
A	0	0	0	0	
B	6	12	5	8	
C	44	88	58	92	
Serum AFP					0.257
<1000 ng/mL	19	38	31	49	
≥1000 ng/mL	31	62	32	51	
Tumor maximal diameter					0.437
<10 cm	17	34	27	43	
≥10 cm	33	66	36	57	
Tumor number					0.703
Single	23	46	26	41	
Multiple	27	54	37	59	
Portal vein tumor thrombus					0.829
Vp0, 1, 2	12	24	17	27	
Vp3, 4	38	76	46	73	
Extrahepatic metastasis					1
yes	11	22	13	21	
no	39	78	50	79	
Child-Pugh class					0.314
A	27	54	38	60	
B	23	46	25	40	
Previous treatment					
TACE	20	40	26	52	
RFA	3	6	5	10	
TARE	1	2	3	6	
Liver resection	2	4	4	8	
Sorafenib	1	2	3	6	
Non-enhancing portion					0.005
<50%	26	52	49	78	
≥50%	24	48	14	22	
Perfusion alteration				0	0.126
no	26	52	42	67	
yes	24	48	21	33	
Targetoid enhancement					0.599
no	44	88	53	84	
yes	6	12	10	16	
Blood product in tumor (T1)					0.852
no	26	52	34	54	
yes	24	48	29	46	
Fatty change in tumor					1
no	44	88	56	89	
yes	6	12	7	11	
**Mean tumor ADC (unit, × 10^-3^ mm^2^/s)**	**1.10 ± 0.29**		**1.10 ± 0.31**		**1**
**Mean liver ADC (unit, × 10^-3^ mm^2^/s)**	**1.54 ± 0.35**		**1.26 ± 0.31**		**0.001**
**Mean spleen ADC (unit, × 10^-3^ mm^2^/s)**	**1.05 ± 0.24**		**1.06 ± 0.19**		**0.757**
**Mean tumor-to-liver ADC ratio**	**0.63 ± 0.10**		**0.98 ± 0.23**		**0.001**
**Mean tumor-to-spleen ADC ratio**	**0.94 ± 0.25**		**1.16 ± 0.27**		**0.001**
Diffusion restriction					0.787
no	8	16	8	13	
yes	42	84	55	87	
Tumor signal on arterial phase					0.662
higher than parenchyma	45	90	56	89	
similar to liver parenchyma	2	4	4	6	
lower than liver parenchyma	3	6	3	5	
Homogenous enhancement				0	0.014
no	6	12	20	32	
yes	44	88	43	68	
Median HAIC session number	5.0 ± 3.3		4.3 ± 3.1		0.297
**Response to HAIC**					**0.006**
** CR+PR**	**21**	**42**	**11**	**17**	
** SD+PD**	**29**	**58**	**52**	**83**	

HAIC, Hepatic arterial infusion chemotherapy; HBV, hepatitis B virus; HCV, hepatitis C virus; BCLC, Barcelona Clinical Liver Cancer; AFP, alpha-fetoprotein; TACE, transarterial chemoembolization; RFA, radiofrequency ablation; TARE, transarterial radioembolization; ADC, apparent diffusion coefficient.

Significant variables between two groups are in bold characters.

### Intrahepatic Response According to the Pretreatment ADC Tumor-to-Liver Ratio

**Figure 2:**
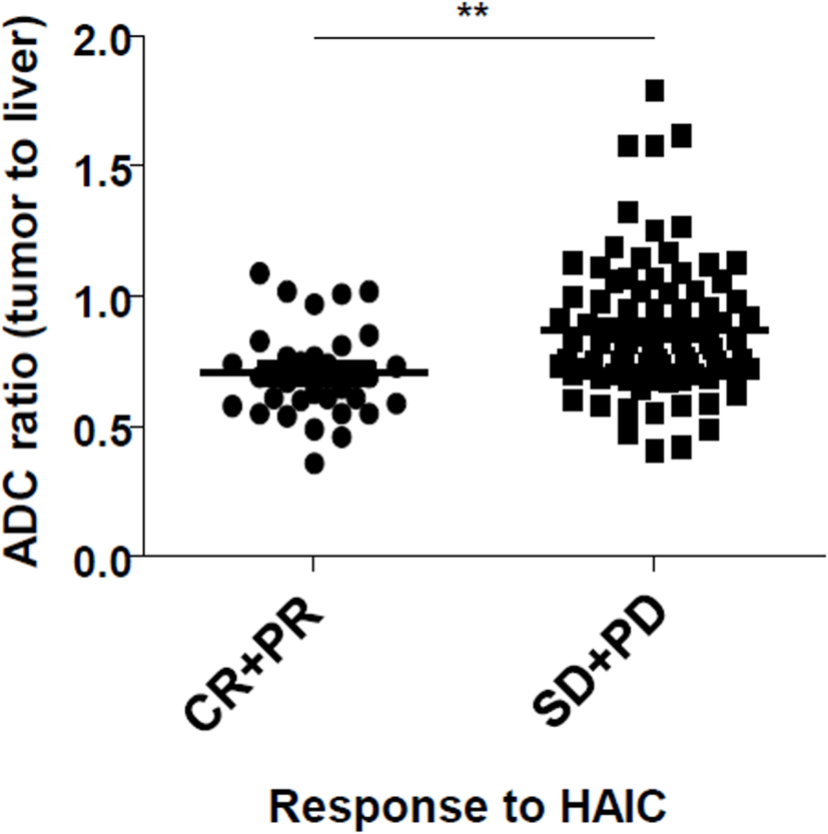
Pretreatment tumor-to-liver ADC ratio according to the response to HAIC. **P < 0.01.

As indicated in **Table 2**, the optimal intrahepatic response to therapy was evaluated based on mRECIST following 2-3 HAIC cycles. In the low group, the number of patients who displayed CR or PR was 21 (42%) and SD or PD was 29 (58%). In the high group, the number of patients who displayed CR or PR was 11 (17%) and SD or PD was 52 (83%). There was a statistical difference between the objective response rate between the low and the high group (*P* = 0.006). **Figure 2** shows that the pretreatment tumor-to-liver ADC ratio of patients with objective responses was significantly lower than that of patients without objective responses (*P* < 0.01). OS were significantly higher in patients with objective responses to HAIC than in those without objective responses (*P* = 0.001 by log-rank test, **Supplementary Figure 2A**). However, there was no significant difference in OS (**Supplementary Figure 2B**) and progression-free survival (**Supplementary Figure 2C**) between the patients with high pretreatment tumor-to-liver ADC ratio and those with low pretreatment tumor-to-liver ADC ratio by log-rank test.

### Factors Affecting Responses to HAIC

**Table 3** delineates the factors affecting the responses to HAIC. Variables included in the logistic regression were as follows: age < 60 years, male sex, maximal tumor diameter < 10 cm, presence of PVTT as Vp0 to Vp2, AFP lower than 1,000 ng/mL, non-enhancing portion of the tumor less than 50%, the presence of perfusion alteration, and the pretreatment tumor-to-liver ADC ratio less than 0.741 (**Table 3**). Among all the factors, the pretreatment tumor-to-liver ADC ratio was the only factor that had a significant effect on the objective responses to cisplatin-based HAIC (odds ratio: 3.217, 95% confidence interval: 1.264–8.187, *P* = 0.014) (**Table 3**).

**Table 3:** Factors affecting the responses to HAIC.

Variables	Logistic regression analysis for objective response	
	*P*	OR (95% CI)
Age (< 60)	0.374	0.643 (0.244–1.699)
Sex (male)	0.423	1.662 (0.479–5.761)
Size (<10 cm)	0.683	1.217 (0.473–3.131)
Portal vein tumor thrombus (Vp0 to Vp2)	0.789	0.862 (0.290–2.566)
AFP (< 1000 ng/mL)	0.962	1.023 (0.404–2.593)
Non-enhancing portion (< 50%)	0.559	0.737 (0.265–2.049)
Perfusion alteration (present)	0.388	0.654 (0.250–1.715)
**Pretreatment tumor-to-liver ADC ratio**	**0.014**	**3.217 (1.264–8.187)**

HAIC, Hepatic arterial infusion chemotherapy; AFP, alpha fetoprotein; ADC, apparent diffusion coefficient; OR, odds ratio; CI, confidence interval.

Significant variable in regression analysis is in bold characters.

**Figure 3:**
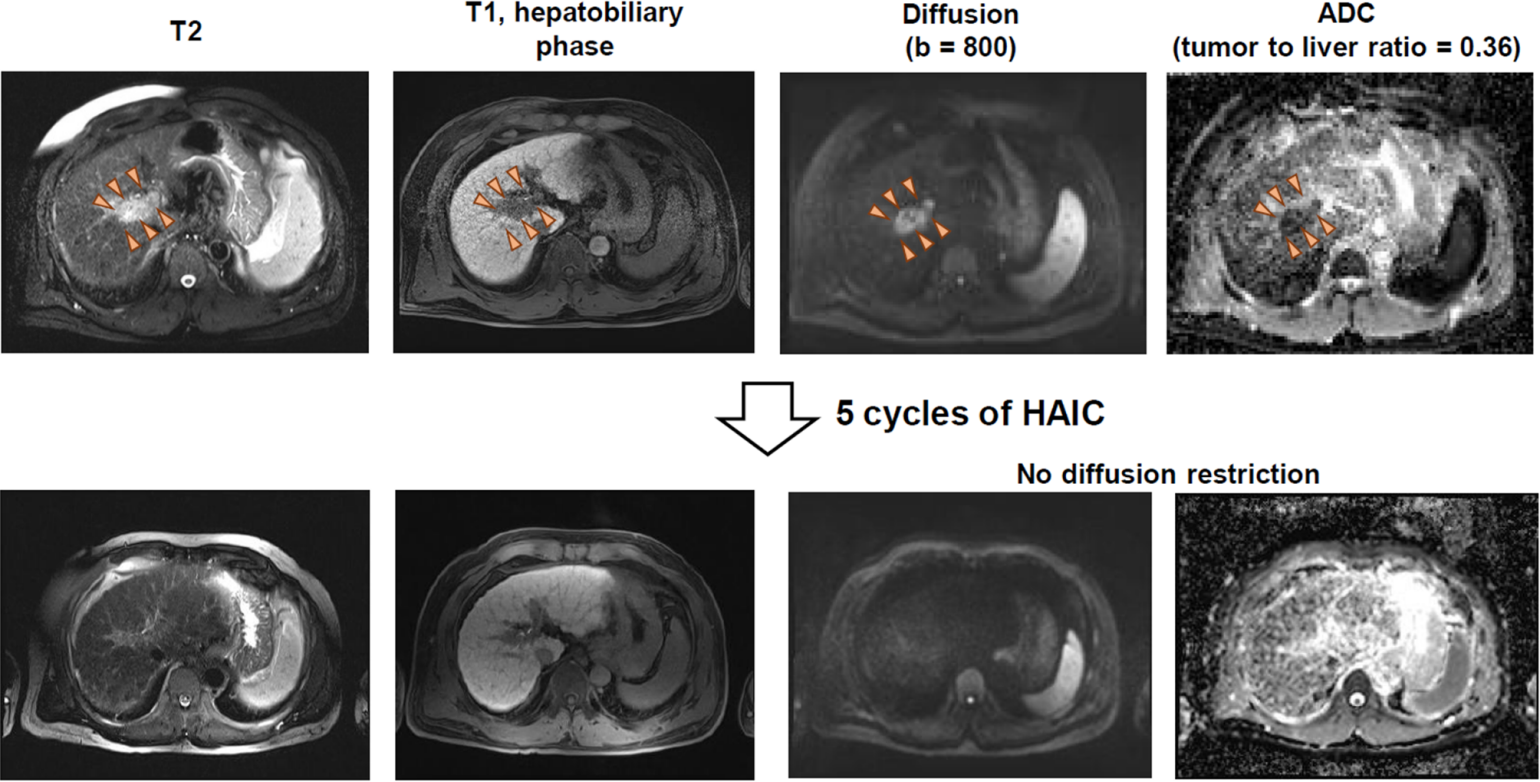
MR imaging of a patient having HCC with strong diffusion restriction and good response to HAIC.

**Figure 3** shows the MR imaging of a representative patient case having HCC with strong diffusion restriction and excellent response to HAIC. For the patient, the main tumor was located in the main portal vein, and the pretreatment tumor-to-liver ADC ratio was 0.36. After 5 cycles of cisplatin-based HAIC, there was no viable tumor with diffusion restriction (**Figure 3**). The patient underwent subsequent liver transplantation after the achievement of CR by HAIC. Explant histology showed no viable tumor and complete pathologic response to HAIC.

## Discussion

Advanced HCC usually shows poor prognosis, with the aim of treatment being limited to extending life and at the same time preserving the hepatic reserve. BCLC stage B or C HCC with high intrahepatic tumor burden has typically been treated with the multikinase inhibitor sorafenib or lenvatinib (21). However, these drugs have been shown to improve survival only slightly. Moreover, when HBV is the cause of HCC, the prevalence of PVTT and more aggressive tumor features is higher than when other etiologies are the causes (2). Recent work by our group has demonstrated that survival outcomes in some advanced HCC cases may be improved dramatically by HAIC because of the substantial reduction of the intrahepatic tumor burden, even in cases with Vp 3/4 PVTT or extrahepatic metastases (2). Therefore, it is critical to identify the patients who will potentially benefit from HAIC. In this study, we suggest that decreased pretreatment tumor-to-liver ADC ratio may be a marker of an objective response to cisplatin-based HAIC. We compared the cutoff value of ADC tumor-to-liver ratio (0.741) in this study with those of prior studies in patients HCC. There were a few studies that described the prognostic significance of tumor-to-liver ADC ratio in HCC. In one study, the mean tumor-to-liver ADC ratio was 1.13 ± 0.63. The study included patients with pathologically confirmed HCC, and the ADC ratio was neither associated with tumor size or differentiation grade. In another study, lower tumor-to-liver ADC ratio (cutoff: 0.820) was a significant factor to predict CK19-positive HCC. Our cutoff level (0.741) is similar with that in the latter study (0.820). Because HAIC is usually performed in patients with advanced HCC, the cutoff value of our study may be lower than those of other studies, reflecting higher cellularity of the tumors.

DWI was used for predicting the responses to various local and systemic therapies in patients with HCC (22). For TACE, a significant increase in the mean ADC of the tumors with a simultaneous reduction in the intra-tumoral enhancement was reported in treated tumors (16, 17). For radioembolization, a previous study reported a modest ADC increases post-treatment (20). For the systemic treatment, a pilot study reported that a significant increase in perfusion fraction was noted in sorafenib responders, although overall ADC was not significantly altered between responders and non-responders (23, 24).

DWI is now used in most of the cancers to predict treatment responses and to distinguish different tumor grades (12). For instance, patients with breast cancer and a low pretreatment ADC tended to respond better to neoadjuvant chemotherapy (2). Recent reports demonstrated that DWI helps distinguishing early HCCs from regenerative nodules in cirrhotic livers (22, 25). Moreover, DWI predicted the pathologic grade of HCC because there was an inverse correlation between tumor grades and ADC values (13, 26, 27). For patients treated with cisplatin-based HAIC, this strategy will also provide benefits to patients. Despite the known chemoresistance of HCC to cytotoxic drugs such as cisplatin (17, 28), there are certainly a group of patients that show dramatic responses to this treatment (29). The reason there is a group of patients who show objective responses to this treatment will be identified when detailed multi-omics analyses are performed. Previous reports demonstrated that downregulated expression of specific genes may render susceptibility to cisplatin in HCC cell lines (17, 28). This suggests that patients with downregulation of these genes in their tumors may show a good response to cisplatin-based HAIC. The molecular-radiologic correlation regarding cisplatin sensitivity in HCC requires further investigation.

There are a number of shortcomings to this study. One shortcoming is that the study was conducted in one institution, so there is a possibility of selection bias. Another shortcoming is the insufficient number of cases recruited. Moreover, liver fibrosis/cirrhosis can lower the ADC values of the liver parenchyma on MRI. Therefore, the pretreatment tumor-to-liver ADC ratio could be affected the degree of liver fibrosis in this study. Moreover, PVTT is not measurable in mRECIST criteria and most of the patients with good responses to HAIC in this study had PVTT, which might have caused the tumor burden measured by mRECIST not to be associated with HAIC responses. A cohort study with a larger number of patients with more stratified analyses should be performed. On the other hand, this is the first study to identify imaging biomarkers of HAIC in advanced HCC. Although comparable analyses were conducted on cases receiving sorafenib treatment during the same period, statistical analyses were not possible because only five of the over 250 cases displayed intrahepatic objective responses following sorafenib treatment.

In a recent clinical trial, lenvatinib was non-inferior to sorafenib in terms of OS in patients with unresectable HCC and caused a considerable decrease in the tumor burden when patients were responsive to the drug (30–32). Therefore, the combined use of lenvatinib plus cisplatin-based HAIC may show the synergistic anti-cancer effects in advanced HCC. A future prospective clinical trial of lenvatinib plus cisplatin-based HAIC vs. lenvatinib only may show promising results in combination treatment. Investigation of the roles of DWI and contrast-enhanced MRI in lenvatinib plus HAIC will also be an area for interesting research.

HCC is a typical example of malignancy associated with nonresolving inflammation (33–35). However, HCC is recognized as an immune-tolerant malignancy (36). Only 14% to 18% of patients who receive pembrolizumab or nivolumab monotherapy demonstrate objective tumor responses (37–39). In HCC, immune heterogeneity is characteristic of larger tumors containing more clones that are resistant to immune checkpoint inhibitors. To overcome this heterogeneity, studies have investigated the synergic benefits of combination therapy for advanced HCC (40). Lenvatinib combined with pembrolizumab or bevacizumab with atezolizumab demonstrated unprecedented objective response rates (41, 42). These reports suggest that the resistance to immune checkpoint inhibitors can be overcome by the combination of drugs with different mechanisms of action. Future studies will demonstrate the role of DWI in predicting the responses of various immune and combination therapies.

In conclusion, our study demonstrated for the first time that patients with unresectable HCC with a pretreatment tumor-to-liver ADC ratio < 0.741 showed a favorable intrahepatic response to HAIC. Therefore, diffusion-weighted MR imaging can play a critical role as a predictor of response to cisplatin-based HAIC in unresectable HCC.

## Data Availability Statement

The original contributions presented in the study are included in the article/**Supplementary Material**. Further inquiries can be directed to the corresponding authors.

## Ethics Statement

Ethical approval was obtained from the Institutional Review Board of Seoul St. Mary’s Hospital (KC19RESI0912). The patients/participants provided their written informed consent to participate in this study.

## Author Contributions

PS: study design, data collection, data analysis, data interpretation, manuscript writing, and manuscript approval. MC: data collection, data analysis, data interpretation, manuscript writing, and manuscript approval. HY, HC, JJ, JC, SY, J-IC, YL, and SB: data interpretation and manuscript approval. All authors contributed to the article and approved the submitted version.

## Funding

This research was supported by the Basic Science Research Program through the National Research Foundation of Korea (NRF) (NRF-2019R1I1A1A01059642 to PS and NRF-2020R1A2C3011569 to SB). This work was also supported by Research Fund of Seoul St. Mary’s Hospital, The Catholic University of Korea (PS).

## Conflict of Interest

The authors declare that the research was conducted in the absence of any commercial or financial relationships that could be construed as a potential conflict of interest.
