# Supplementary figures and images for: Diffusion-Weighted Magnetic Resonance Imaging in Hepatocellular Carcinoma as a Predictor of a Response to Cisplatin-Based Hepatic Arterial Infusion Chemotherapy
Source: Front Oncol. 2020 Nov 19;10:600233. doi: 10.3389/fonc.2020.600233 (PMC7711158; doi:10.3389/fonc.2020.600233)

## Supplementary Figure 1

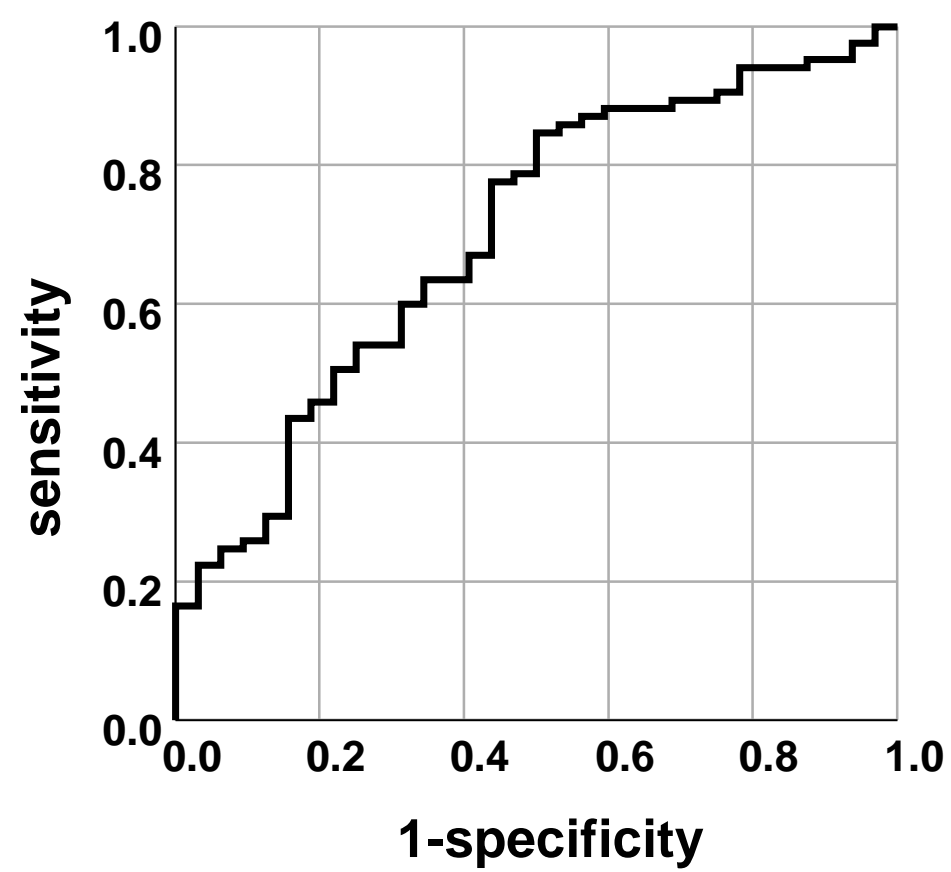

AUROC: 0.704

Cutoff value of tumor-to-liver ADC: 0.741

Supplementary Figure 2

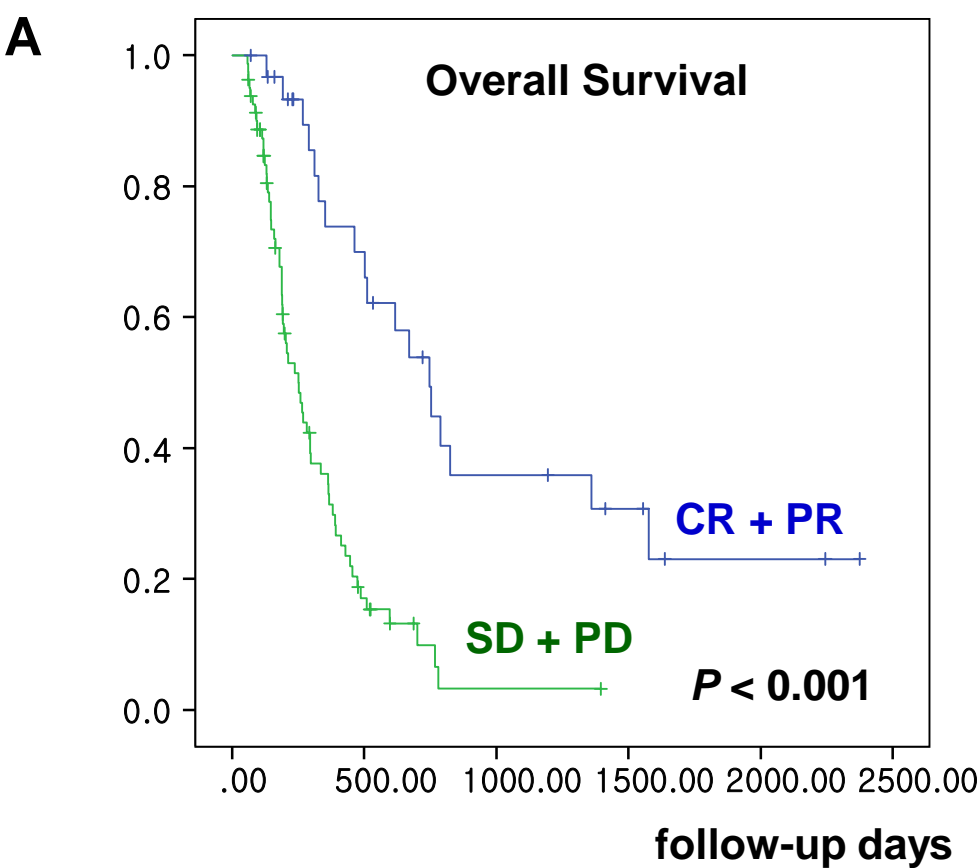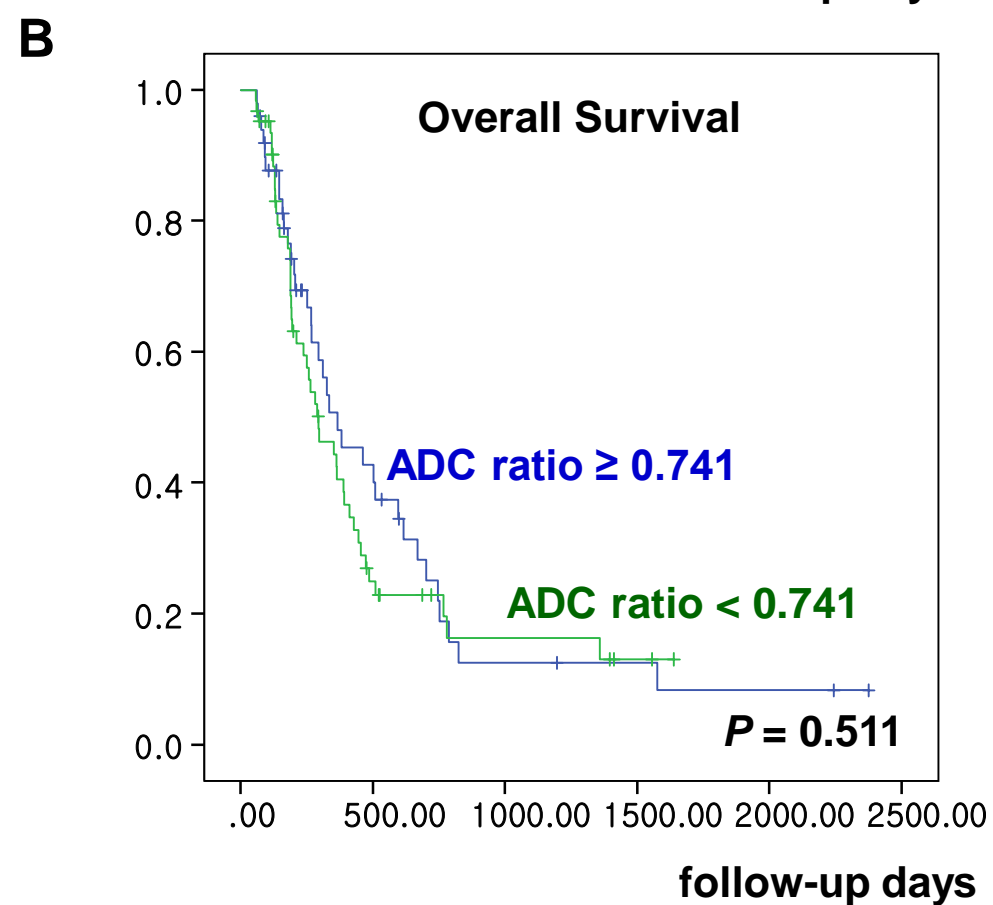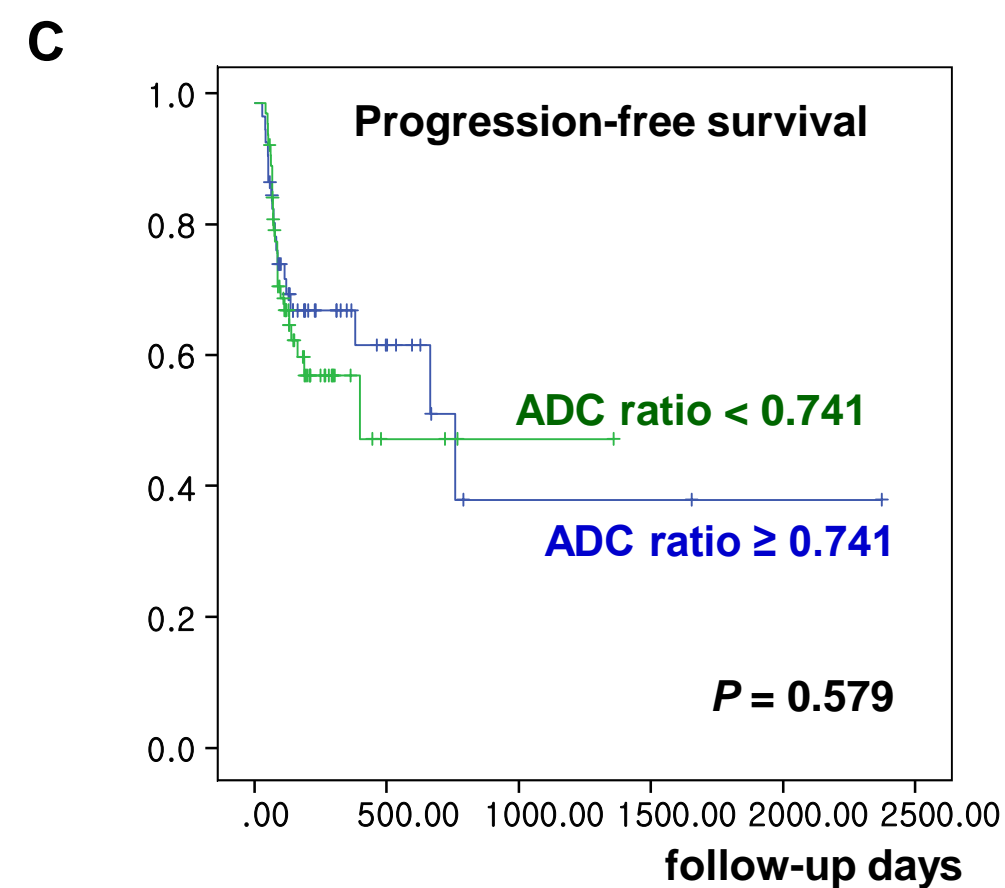

Supplement: Supplementary file 1 [file DataSheet_1.pdf]
